# Interfacial Water Structure as a Descriptor for Its Electro-Reduction on Ni(OH)_2_-Modified Cu(111)

**DOI:** 10.1021/acscatal.1c02673

**Published:** 2021-08-04

**Authors:** Andrea Auer, Francisco J. Sarabia, Daniel Winkler, Christoph Griesser, Víctor Climent, Juan M. Feliu, Julia Kunze-Liebhäuser

**Affiliations:** † Institute of Physical Chemistry, University of Innsbruck, Innrain 52c, Innsbruck 6020, Austria; ‡ Instituto Universitario de Electroquímica, Universidad de Alicante, Carretera San Vicente del Raspeig s/n, E-03690 San Vicente del Raspeig, Alicante, Spain

**Keywords:** hydrogen evolution reaction, Cu single crystals, Ni(OH)_2_ modification, *in situ* electrochemical scanning tunneling microscopy, potential of maximum entropy, interfacial water reorganization, laser-induced temperature jump

## Abstract

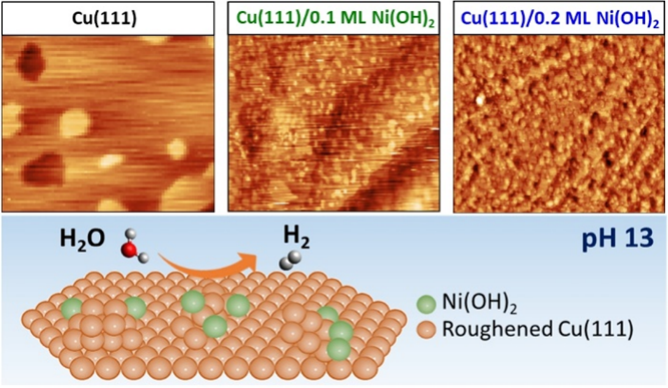

The hydrogen evolution reaction (HER) has been crucial for the development of fundamental knowledge on electrocatalysis and electrochemistry, in general. In alkaline media, many key questions concerning pH-dependent structure–activity relations and the underlying activity descriptors remain unclear. While the presence of Ni(OH)_2_ deposited on Pt(111) has been shown to highly improve the rate of the HER through the electrode’s bifunctionality, no studies exist on how low coverages of Ni(OH)_2_ influence the electrocatalytic behavior of Cu surfaces, which is a low-cost alternative to Pt. Here, we demonstrate that Cu(111) modified with 0.1 and 0.2 monolayers (ML) of Ni(OH)_2_ exhibits an unusual non-linear activity trend with increasing coverage. By combining *in situ* structural investigations with studies on the interfacial water orientation using electrochemical scanning tunneling microscopy and laser-induced temperature jump experiments, we find a correlation between a particular threshold of surface roughness and the decrease in the ordering of the water network at the interface. The highly disordered water ad-layer close to the onset of the HER, which is only present for 0.2 ML of Ni(OH)_2_, facilitates the reorganization of the interfacial water molecules to accommodate for charge transfer, thus enhancing the rate of the reaction. These findings strongly suggest a general validity of the interfacial water reorganization as an activity descriptor for the HER in alkaline media.

## 1 Introduction

The hydrogen evolution reaction (HER) and its electrocatalytic characteristics are central to a number of technologically important processes in the development of hydrogen-based energy sources, a pivotal alternative to fossil fuels. The HER is an ideal model reaction and hence of fundamental scientific importance since it helped to develop basic concepts of modern electrocatalysis.^1−3^ While in acidic media it is generally accepted that the hydrogen binding energy (HBE) is the sole activity descriptor, there is much debate on the energetics and kinetics of the alkaline HER.^4^ Markovic and co-workers have continuously pointed out that in alkaline solutions there is a second descriptor: the binding and energetics of adsorbed hydroxyl species (OH_ad_).^5−9^ In previous works, they have shown the importance of favorable OH adsorption, which seems to enhance the rate of water dissociation by lowering the energy barrier for H_2_O activation through a metal–OH_ad_–H_2_O complex.^5,7,10^ It is further stated that by incorporating more oxophilic sites, which offer a stronger OH binding, catalysts show an enhanced activity toward the HER in alkaline media.^5,8,10^ This led to the idea of modifying platinum (Pt), the state-of-the-art catalyst for the HER due to its optimal HBE, with more oxophilic species, i.e., Ni(OH)_2_ ad-islands.^6^ The resulting bifunctional catalyst shows a significantly improved activity, where, mechanistically, the randomly distributed Ni(OH)_2_ clusters promote the dissociation of water and thereby enhance the formation of adsorbed hydrogen (H_ad_) intermediates, which are collected by the Pt substrate and subsequently recombined to form H_2_.^6^ In accordance with these observations, it has been shown on Pt(111) decorated with different 3d transition metal hydroxides that the affinity of the oxophilic sites to OH_ad_ should neither be too strong nor too weak to promote the HER activity in alkaline media.^7^

In addition to this bifunctional mechanism, it was suggested by the groups in Alicante and Leiden that Ni(OH)_2_ promotes the HER by lowering the energy barrier associated with the reorganization of the interfacial water network, which then allows for an easier charge transfer through the double layer.^11,12^ Through modification of Pt with Ni(OH)_2_ islands, the electric field strength decreases significantly due to a negative shift of the potential of zero free charge (pzfc) or the potential of maximum entropy (pme).^11,12^

The generality of the promoting effect of adding Ni(OH)_2_ to a catalyst’s surface has been clearly shown for a variety of metals, including IB group metals (i.e., Cu, Ag, and Au).^8^ The validity of the proposed descriptors, however, i.e., the bifunctionality given through oxophilic sites for favorable OH_ad_ energetics on the one hand and the strength of the interfacial electric field influencing the energetic barrier associated with water reorganization during proton/hydroxide transfer on the other hand, has only been confirmed for Pt-group metal-based electrocatalysts. Cu is a low-cost alternative to Pt-group metals, with a uniquely low tendency toward hydride formation in long-term operations.^8^ In this work, we therefore show that modifying Cu(111) electrodes with low amounts of Ni(OH)_2_ leads to an unexpected non-linear activity increase with increasing coverage. Our electrochemical measurements reveal that while adding small amounts of only 0.1 ML of Ni(OH)_2_ to the Cu surface does not lead to a significant (kinetic) enhancement of the HER in the alkaline electrolyte, the activity for 0.2 ML of Ni(OH)_2_ is enhanced by a factor of 13 relative to bare Cu(111). *In situ* electrochemical scanning tunneling microscopy (EC-STM) reveals that the morphology of the Cu surface drastically changes upon Ni(OH)_2_ deposition, which leads to an increased roughness. While the mean-square roughness of the surface scales linearly with increasing Ni(OH)_2_ surface concentration, we find its impact on the interfacial water layer structure to be nonuniform. Laser-induced potential transient measurements evidence a potential-dependent water orientation, where a highly disordered interfacial water layer exists close to the onset of the HER for 0.2 ML of Ni(OH)_2_ only. Water dissociation is thus facilitated by a lower energetic barrier for charge movement across the electrochemical double layer. This molecular-level understanding is an essential step toward unraveling the complexity and determining the general validity of activity descriptors for the HER in alkaline media and enables the development of a more complete structure–activity relation for Cu composite materials.

## 2 Results and Discussion

**Figure 1:**
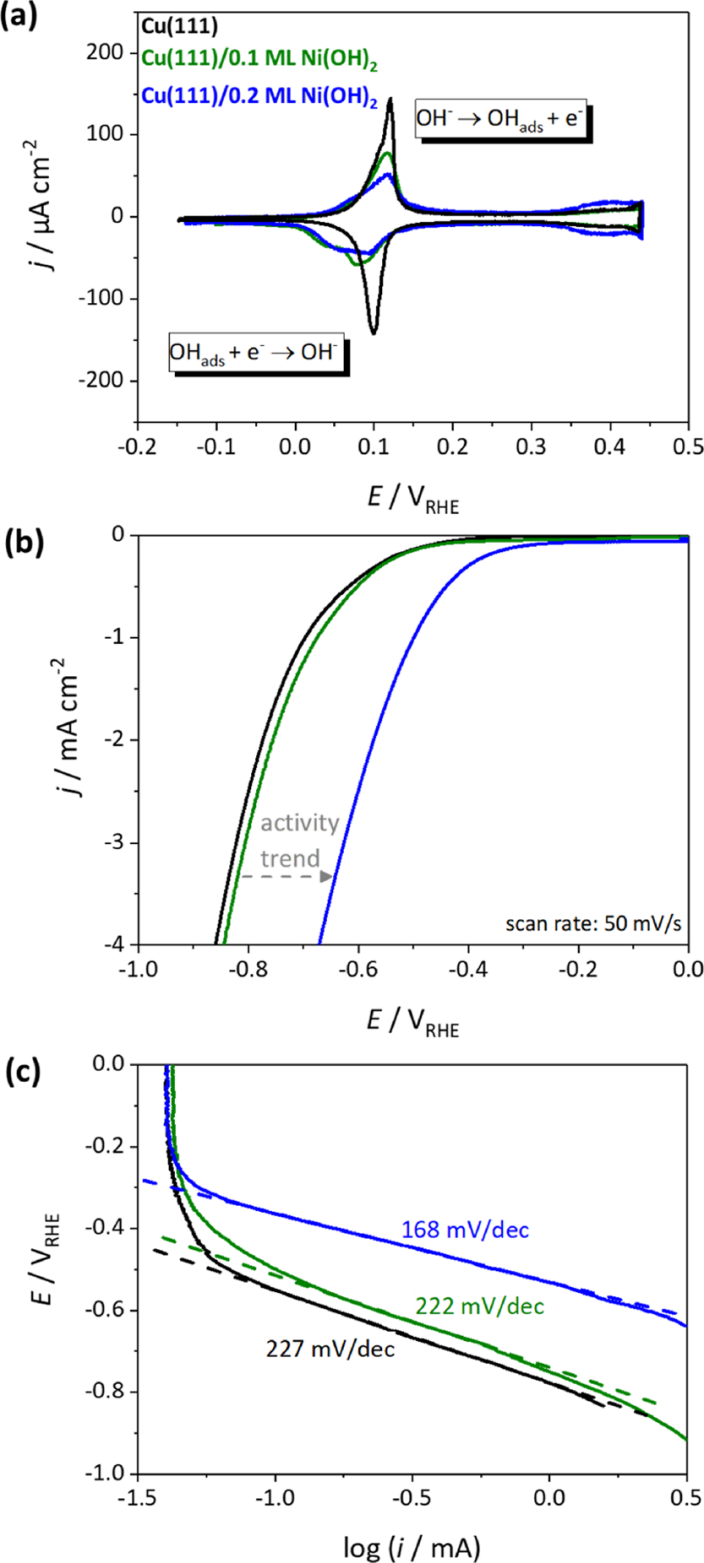
Electrochemical and electrocatalytic properties of Ni(OH)_2_-modified Cu(111) electrodes. (a) Fingerprint cyclic voltammograms (CVs) for Cu(111) (black), Cu(111) modified with 0.1 ML of Ni(OH)_2_ (green), and Cu(111) with 0.2 ML of Ni(OH)_2_ (blue) in 0.1 M NaOH with a scan rate of 50 mV/s. (b) HER activities for Cu(111), Cu(111)/0.1 ML of Ni(OH)_2_, and Cu(111)/0.2 ML of Ni(OH)_2_. (c) Corresponding Tafel plots for the HER calculated from the measurements recorded in the flow cell at 2 mV/s (see Figure 2a).

To determine the effect of Ni(OH)_2_ deposition on the electrochemical behavior of Cu(111), cyclic voltammetry was employed. All potentials are given with respect to the reversible hydrogen electrode (RHE). Figure 1a shows the voltammetric profiles of Cu(111) with different Ni(OH)_2_ coverages, i.e., 0.1 and 0.2 ML, in 0.1 M NaOH. The blank voltammogram without Ni(OH)_2_ is also shown as reference. A distinct peak pair, corresponding to the adsorption and desorption of hydroxide (OH) on Cu(111), with maxima at around 0.12 V_RHE_,^13,14^ can be observed in the blank voltammogram. This peak significantly decreases in current density with increasing Ni(OH)_2_ coverage. The clearly defined OH adsorption feature in the pseudocapacitive region of the Cu(111) cyclic voltammogram (CV) enables us to estimate the degree of blockage of the Cu surface and therefore the apparent Ni(OH)_2_ coverage from the remaining charge (see Figure S1 and Table S1 in the Supporting Information (SI)). Although there are uncertainties and assumptions made in the used coverage determination, clearly the charge of the fingerprint OH adsorption feature relatively decreases with increasing Ni(OH)_2_ surface concentration.

**Figure 2:**
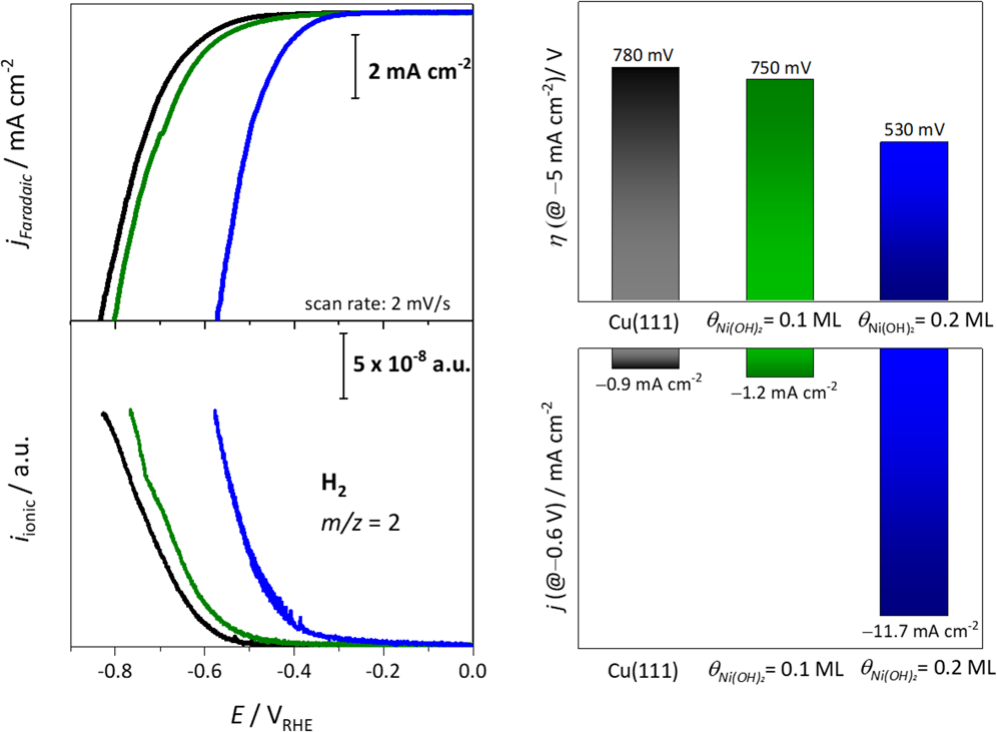
(a) Polarization curves for Cu(111) (black), Cu(111)/0.1 ML of Ni(OH)_2_ (green), and Cu(111)/0.2 ML of Ni(OH)_2_ (blue) with the corresponding *m*/*z* = 2 signal for H_2_. Comparison between the activities toward the HER expressed as (b) the overpotential at a current density of −5 mA cm^–2^ and (c) the current density at −0.6 V_RHE_.

The electrocatalytic properties of the Ni(OH)_2_-modified Cu(111) electrodes toward the HER in alkaline media are depicted in Figure 1b. Interestingly, the HER activity is non-linearly enhanced with increasing Ni(OH)_2_ coverage. While for Cu(111)/0.1 ML of Ni(OH)_2_ only a small promoting effect can be observed, the activity increases significantly for Cu(111)/0.2 ML of Ni(OH)_2_. The commonly accepted mechanism of the HER in alkaline solutions is typically described through a combination of three steps: the Volmer step involves water dissociation (H_2_O + M + e^–^ ⇌ M-H_ad_ + OH^–^) and is followed by either the Tafel step (2M-H_ad_ ⇌ 2M + H_2_) or the Heyrovsky step (H_2_O + M-H_ad_ + e^–^ ⇌ M + H_2_ + OH^–^), where M stands for any metal acting as a catalyst.^4,15^ Although a rigorous kinetic analysis of the HER is generally difficult and lies beyond the scope of the present work, Tafel plots are depicted in Figure 1c for comparison of their slopes. To estimate those Tafel slopes, polarization curves at 2 mV/s were recorded in a flow cell (see Figure 2a). We find that both the bare Cu(111) and Cu(111)/0.1 ML of Ni(OH)_2_ exhibit the same high Tafel slope of 222–227 mV/dec, whereas it decreases to around 168 mV/dec for a Ni(OH)_2_ coverage of 0.2 ML. Unexpectedly, this implies that, by decorating Cu(111) with only 0.1 ML of Ni(OH)_2_, no kinetic enhancement is obtained, while for slightly higher coverages of 0.2 ML of Ni(OH)_2_, the significantly lower Tafel slope indicates much faster kinetics. For a more accurate and more quantitative determination of the electrocatalytic behavior of the Ni(OH)_2_-modified Cu(111), online differential electrochemical mass spectrometry (DEMS) was employed in a flow cell configuration. The results are shown in Figure 2.

Figure 2a shows the polarization curves in the potential range of the HER with a scan rate of 2 mV/s and the corresponding mass-to-charge signal (*m*/*z* = 2) for H_2_. The unusual non-linear activity enhancement for 0.2 ML of Ni(OH)_2_ on Cu(111) is sustained when the HER activities are considered as either the overpotential (η) or the current density. The overpotential at a current density of −5 mA cm^–2^ is lowered by 30 mV through the presence of 0.1 ML and by 150 mV through 0.2 ML of Ni(OH)_2_ (see Figure 2b). At −0.6 V_RHE_, the HER current density is enhanced by factors of 1.4 and 13 relative to the bare Cu(111) for Ni(OH)_2_ coverages of 0.1 and 0.2 ML, respectively (see Figure 2c). This is in striking contrast to Ni(OH)_2_-modified Pt(111), where a linear increase in activity with increasing coverage of Ni(OH)_2_ was observed.^12^

To understand this clear difference between Ni(OH)_2_-modified Cu(111) and Pt(111), quasi *in situ* X-ray photoelectron spectroscopy^16^ was employed to evaluate the chemical composition of the Cu(111)/Ni(OH)_2_ electrodes.

**Figure 3:**
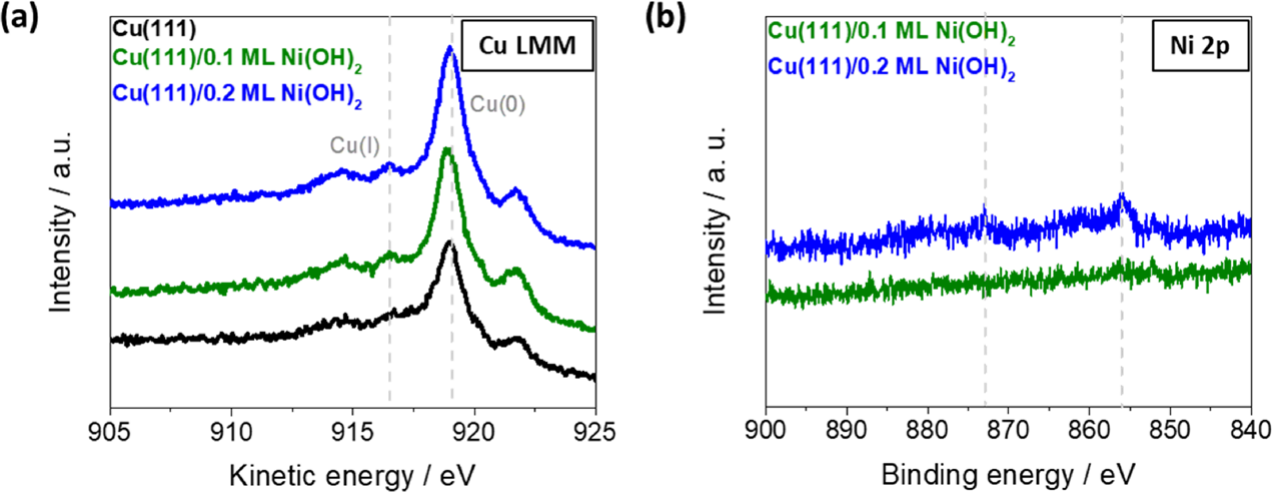
Quasi *in situ* XPS: (a) Cu LMM Auger signals for Cu(111) (black), Cu(111)/0.1ML of Ni(OH)_2_ (green), and Cu(111)/0.2 ML of Ni(OH)_2_ (blue), and (b) XPS spectra of the Ni 2p region for Cu(111)/0.1ML of Ni(OH)_2_ (green) and Cu(111)/0.2 ML of Ni(OH)_2_ (blue). All spectra are acquired with a take-off angle of 60° between the Cu(111) surface normal and the analyzer.

Figure 3a shows the Cu LMM Auger spectra for each electrode, which are used to determine the different Cu oxidation states via the relative intensities of the peaks.^10,17^ The relative intensities of the highest peak at a kinetic energy of ∼919 eV, attributed to Cu(0) species,^18^ and the peak at ∼917 eV, ascribed to Cu (I) species,^18^ remain constant for all samples (Cu(0) ≫ Cu(I)). This strongly suggests that, by adding Ni(OH)_2_ to Cu(111), the Cu surface remains mostly metallic with an oxidation state of Cu(0). Interestingly, this differs from previous findings where a clear correlation between higher amounts of Cu(I) species, and hence a higher oxophilicity of differently treated Cu surfaces, and the enhancement of the HER activity was proposed.^10^ The Ni 2p region, shown in Figure 3b for the modified Cu(111) electrodes, reveals the presence of low amounts of oxidized Ni on the surface. The observed small peaks with binding energies of around 856 and 873 eV, corresponding to Ni 2p_3/2_ and Ni 2p_1/2_, with a spin-energy separation of 17 eV, are consistent with the literature values reported for Ni(OH)_2_.^19^ Our XPS data thus clearly refute that the observed non-linear increase in activity with increasing Ni(OH)_2_ coverage is related to a difference in the oxidation state of the Cu surface (i.e., higher amounts of Cu(I) species). We therefore suspect the unusual activity trends to result from a more fundamental difference between Cu and Pt. Cu as a coinage metal has been repeatedly found to dynamically restructure upon anion adsorption,^20−24^ under reaction conditions (e.g., hydrogen evolution,^25,26^ CO_(2)_ reduction,^27−29^ or CO oxidation^30^), and even at its pzfc.^14^ This gives rise to an inherently much lower stability of Cu surfaces compared to, e.g., Pt. While decorating Pt(111) with Ni(OH)_2_ was found to result in well-separated, randomly distributed Ni(OH)_2_ clusters,^5,6^ it is doubtful that the Cu surface stays intact upon Ni(OH)_2_ deposition due to its much lower cohesive energy (3.5 eV vs 5.84 eV for Pt).^31^

To assess this hypothesis, we performed *in situ* EC-STM to investigate the structural and morphological effects of Ni(OH)_2_ modification on Cu(111).

**Figure 4:**
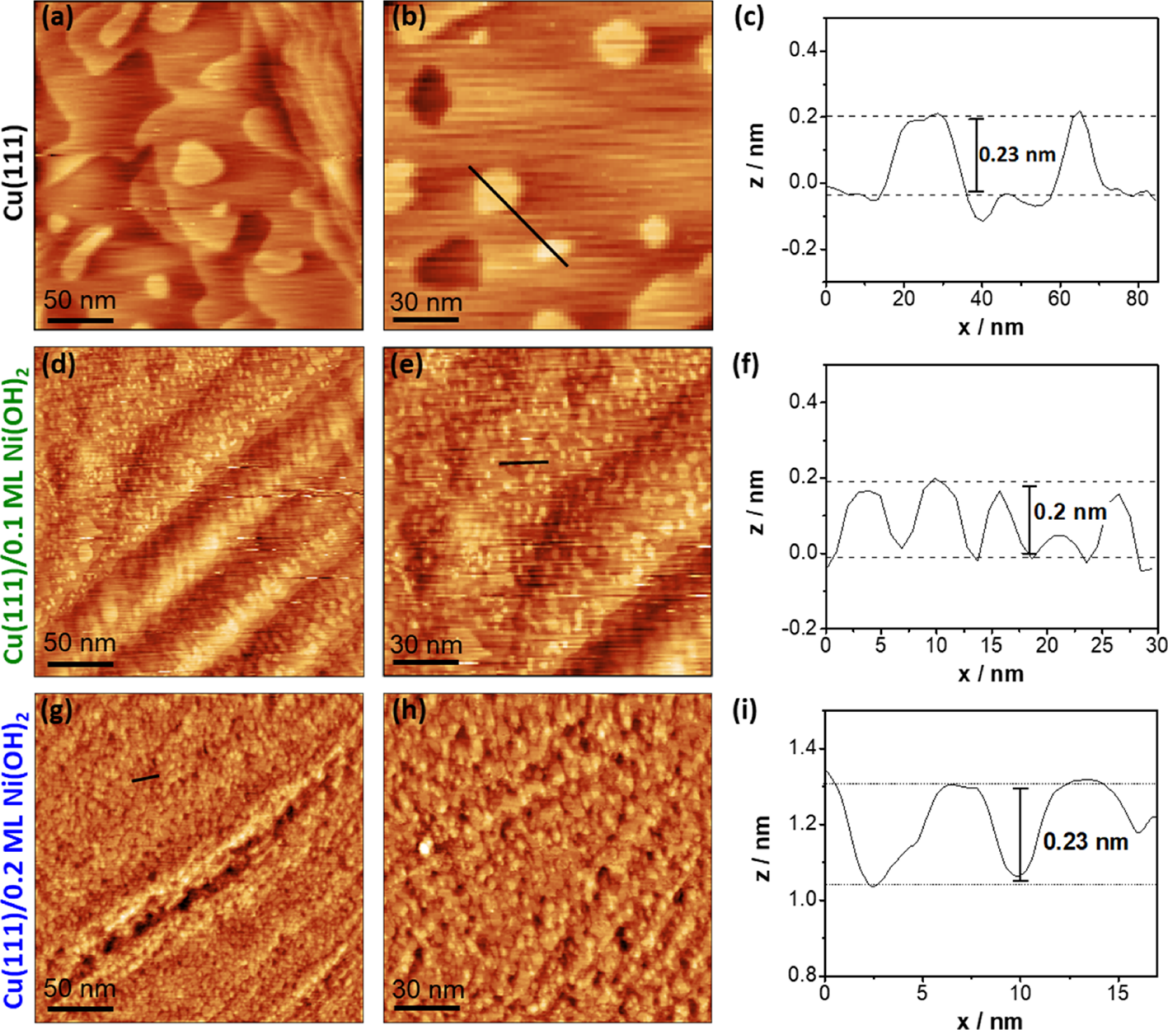
*In situ* EC STM images of (a, b) metallic Cu(111), (d, e) Cu(111)/0.1 ML of Ni(OH)_2_, and (g, h) Cu(111)/0.2 ML of Ni(OH)_2_ at −0.05 V_RHE_ in 0.1 M NaOH. (c, f, i) Corresponding line profiles for Cu(111), Cu(111)/0.1 ML of Ni(OH)_2_, and Cu(111)/0.2 ML of Ni(OH)_2_. Image sizes are (250 × 250) nm^2^ (first column) and (150 × 150) nm^2^ (second column). *I*_tip_ = 1 nA and *E*_tip_ = 0.25 V_RHE_.

As shown in Figure 4a–c, STM imaging of metallic Cu(111) in 0.1 M NaOH reveals that the reduced surface exhibits flat terraces with relatively large ad- and vacancy islands (∼20–50 nm) with a monoatomic step height of approximately 0.22 nm. Adding 0.1 ML of Ni(OH)_2_ leads to the formation and nucleation of additional and much smaller (∼5 nm) Cu ad-islands, separated by monolayer steps (see Figure 4d,e), which can be deduced from the line profile depicted in Figure 4f. This suggests that the Cu(111) surface restructures upon the deposition of low amounts of Ni(OH)_2_. This disintegration of the Cu(111) surface is even more enhanced for a coverage of 0.2 ML of Ni(OH)_2_, where the island density is significantly increased (Figure 4g,h). For 0.1 ML of Ni(OH)_2_, the Cu ad-islands are well separated revealing the underlying terrace structure, whereas the Cu(111) surface is dominated by an almost granular-like morphology after adding 0.2 ML, conforming to a 3D roughening, while still exhibiting the thermodynamic shape and monoatomic step height of Cu ad-islands (Figure 4i). It is well known that Cu surfaces contain mobile ad-atoms (Cu_ad_) detached from undercoordinated sites,^32^ which become increasingly mobile upon adding a surfactant, i.e., CO. This leads to a clustering or roughening of the Cu surface at both the solid/liquid^30^ and solid/gas interfaces.^31,33,34^ Similar to the CO-induced roughening of the Cu surface, it is plausible that Ni(OH)_2_ binds to low-coordinated Cu atoms forming mobile Ni(OH)_2_–Cu_ad_ complexes, which may migrate over the surface and subsequently aggregate to form Cu ad-islands, whose size depends on the Ni(OH)_2_ concentration. Ni(OH)_2_, although not identifiable in the STM images, clearly stabilizes the roughened Cu(111) surface, e.g., through lowering of the step edge formation energy. The observed restructuring induced by the deposition of small amounts of Ni(OH)_2_ and the inherent increase in low-coordinated Cu sites can help to rationalize the observed increase in activity toward the HER. Interestingly, a closer evaluation of representative statistical quantities of the surfaces, in particular, the mean square roughness, with 61, 112, and 181 pm for Cu(111), Cu(111)/0.1 ML of Ni(OH)_2_, and Cu(111)/0.2 ML of Ni(OH)_2_, respectively, shows a rather linear increase with increasing coverages (Figure S2 and Table S2). The non-linear trend in activity enhancement with increasing amounts of Ni(OH)_2_ seems therefore unlikely to result from purely morphological effects, i.e., the roughness factor. However, the possibility that the number of step sites at the restructured Cu(111) surface is not linearly proportional to the determined mean-square roughness and, thus, the contribution of a difference in activities of step and terrace sites to the observed activity trend cannot be excluded.

While the STM data strongly suggest that a clear differentiation between the active sites represented by 3D Ni(OH)_2_ clusters and the bare metal substrate, as observed for modified Pt(111) electrodes, cannot be easily applied to Ni(OH)_2_-modified Cu(111) due to the disintegration of the substrate morphology, the question remains whether we can find a descriptor that allows rationalizing the non-linear activity increase with increasing coverage. Therefore, we performed laser-induced temperature jump experiments, which can lead to unraveling the impact of the interfacial water structure reorganization on the rate of the HER in alkaline media.^11,12^

**Figure 5:**
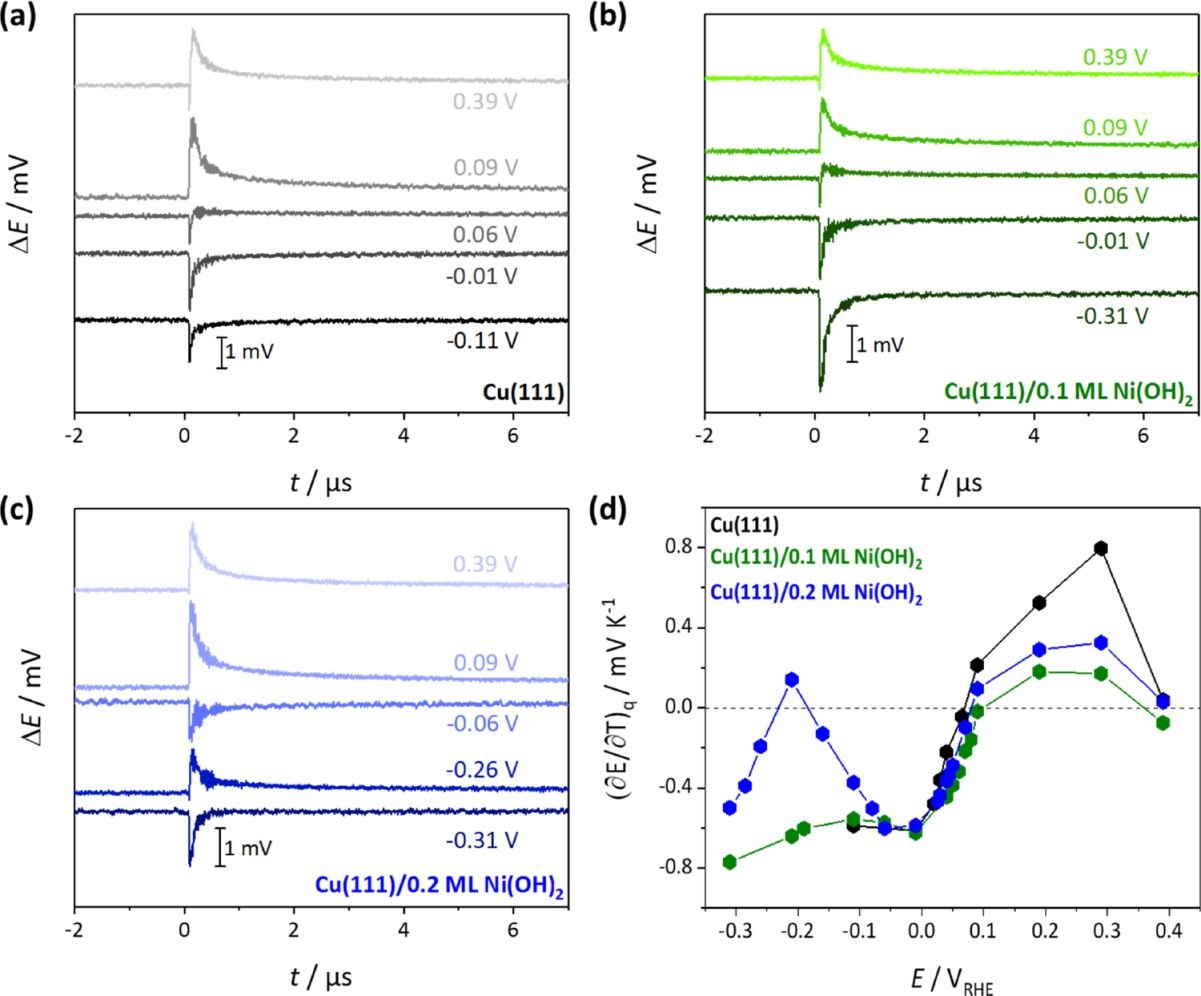
Laser-induced potential transients for (a) the bare Cu(111), (b) Cu(111)/0.1 ML of Ni(OH)_2_, and (c) Cu(111)/0.2 ML of Ni(OH)_2_ at selected potentials. (d) Thermal coefficients for the electrodes (a–c).

Figure 5a–c shows selected laser-induced potential transients for Cu(111) and Cu(111) with the two different Ni(OH)_2_ coverages. The corresponding 3D laser transient plots for Cu(111)/0.1 ML of Ni(OH)_2_ and Cu(111)/0.2 ML of Ni(OH)_2_ are shown in Figure S3 for comparison. We observe, for all investigated electrodes, that at sufficiently high potentials (between 0.35 and 0.1 V_RHE_), the transients are positive, suggesting that the electric field points away from the surface, and the interfacial water molecules are oriented with the oxygen toward the metal. At around 0.07–0.09 V_RHE_, coinciding with the onset of the OH adsorption peak, the transients change their sign to negative, as was previously shown for bare Cu(111) electrodes, which suggest that below this potential, a negative free charge resides at the surface.^14,35^ This change of sign is generally attributed to the position of the pme and a turn-over of the water ad-layer. At the pme, water molecules at the interface distribute randomly, and there is no net dipolar contribution to the electrode potential. It is therefore intimately linked to the pzfc since water dipoles orient mainly according to the electric field at the surface.^36,37^ Here, the pme slightly shifts toward more positive potentials upon Ni(OH)_2_ deposition, exhibiting a trend in the order Cu(111) < 0.2 ML of Ni(OH)_2_ < 0.1 ML of Ni(OH)_2_. At even more negative applied potentials of around −0.2 V_RHE_, the transients for Cu(111)/0.2 ML Ni(OH)_2_ become positive again in a very narrow potential range close to the onset of the HER before flipping over to negative once more. This means that two changes of the sign from negative to positive can be identified, which consequently shows that two potentials of maximum entropy for double layer formation exist, a phenomenon that was previously found for stepped Pt surfaces.^38^ Also related to this phenomena, non-monotonous charge variations on Pt(111) were explained by an electrostatic model of the double layer.^39^ At even lower potentials, the laser transients are all negative, which means that the average water molecule orientation is with the hydrogen toward the metal surface. Introducing a Ni(OH)_2_ surface concentration of 0.2 ML to the Cu(111) surface, however, causes a significant decrease in the overall intensity of these transients in the lowest experimentally accessible potential region (see Figure 5a–c and Figure S3). This evidences a decrease in the structural order of the interfacial water network close to the onset of the HER for Cu(111)/0.2 ML of Ni(OH)_2_.

To more quantitatively analyze the laser-induced potential transients, the thermal coefficients of the potential drop across the double layer (∂*E*/∂*T*) were calculated from linearization of the transients (see description in the Supporting Information and Figure S4 for more details) and are depicted in Figure 5d. Note that the coefficients have been corrected to account for the contribution of the thermodiffusion potential (approximately −0.43 mV K^–1^),^40^ which cannot be neglected due to the high mobility of OH^–^ ions (Figure S5). In the potential region negative of OH adsorption (< 0 V_RHE_), only a very small decrease in the thermal coefficient (∂*E*/∂*T*), which can be interpreted in terms of a decrease in the electric field strength,^11,36^ between Cu(111) and Cu(111)/0.1 ML Ni(OH)_2_ is observed. For coverages of 0.2 ML of Ni(OH)_2_ on the other hand, we find a peak-shaped potential dependence of the thermal coefficient with a maximum at around −0.2 V_RHE_, in agreement with three turn-overs of the water layer. This is perfectly consistent with the existence of two pme values (see Figure S6). The appearance of a second pme was previously ascribed to the existence of different local values of pme on terrace and step sites. This could be caused by a preferential orientation of water on step sites with the oxygen toward the metal due to either a chemical preference or charge–dipole interactions because of a locally induced positive free charge at the steps in the corresponding potential region, resulting from a lower local value of pzfc.^38^ In the case of Cu(111)/Ni(OH)_2_, a similar explanation seems plausible. For coverages of only 0.1 ML of Ni(OH)_2_, where we also observe an increase in Cu ad-island concentration, the step density might be too low for the appearance of a second pme, considering that the transients reflect the response of the whole surface. However, adding 0.2 ML of Ni(OH)_2_ leads to a greater extent of roughening, where the surface consists of 3D Cu ad-islands and holes. The roughness reaches a threshold where the local reorientation of water at the step sites triggers the turn-over of a significant fraction of the entire water ad-layer. In addition, the Ni(OH)_2_ is most likely situated at the step edges as suggested by the EC-STM images, which consequently increases their oxophilicity, and thus, the specific orientation of water molecules with the oxygen toward the metal is even more likely. Therefore, the inherent change in morphology of the Cu surface, which happens spontaneously upon deposition of 0.2 ML of Ni(OH)_2_, leads to a higher disorder in the interfacial water structure at potentials close to the onset of the HER rationalizing the non-linear increase in electrocatalytic activity with increasing coverage. Direct comparison of the above presented LIPT results with the thermal coefficients of Pt(111) electrodes modified with various low amounts of Ni(OH)_2_,^12^ where the electric field strength decreases linearly with increasing Ni(OH)_2_ coverage, can clearly rationalize the significant difference between the non-linear and linear HER activity enhancements of Ni(OH)_2_-modified Cu(111) and Pt(111), respectively. Furthermore, it was previously suggested that both the adsorption strength and the amount of adsorbed OH play a crucial role in the alkaline HER.^4,41^ The addition of Ni(OH)_2_ and thus oxophilic sites can therefore not only play a part in the inversion of the potential transients at low potentials, i.e., the observed second pme at low potentials, but the consequent increase in adsorbed OH, in the form of Ni(OH)_2_ species, can also chemically contribute to the HER activity enhancement. It is therefore likely that both the water dissociation, i.e., the cleavage of the water O–H bond via the bifunctional mechanism, as well as the interfacial electric field strength, and thus, an efficient transport of reactants through the double layer are largely influenced by the presence of Ni(OH)_2_. Both effects can simultaneously contribute to the non-linear activity enhancement. Remarkably, the effect of the second pme is confined to a very narrow potential range between −0.18 and −0.25 V_RHE_. At more negative potentials, where the transients turn negative again, a decrease in the potential dependence of the thermal coefficient is recorded (Figure 5d). Since it has been recently found that Cu(111) reconstructs as a consequence of the change in free charge on the surface,^14^ we performed further *in situ* EC-STM imaging for Cu(111)/0.2 ML of Ni(OH)_2_ to examine possible further structural changes below the second pme.

**Figure 6:**
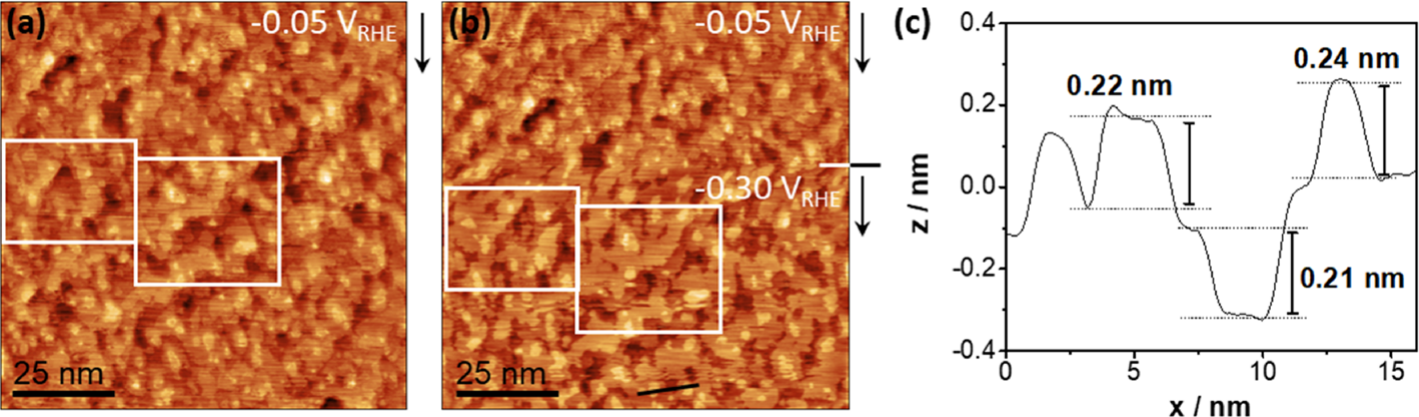
EC-STM visualization of morphological and structural changes of Cu(111)/0.2 ML of Ni(OH)_2_ during a potential step from −0.05 (a) to −0.30 V_RHE_ (b). (c) Corresponding line profile of the surface after the potential step at the position marked by the black line in panel (b). Images size = (100 × 100) nm^2^. *I*_tip_ = 1 nA and *E*_tip_ = 0.25 V_RHE_. Black arrows mark the slow scan direction. Identical positions on the surface before and after the potential step are marked with white squares.

Figure 6 shows a potential step from −0.05 to −0.30 V_RHE_. The lower potential lies in the region of the decrease in electric field strength. The Cu(111) electrode surface undergoes a smoothening process upon this potential step, where larger terraces with rounded edges are reformed. This process greatly resembles the so-called “electrochemical annealing”, which was previously reported for, e.g., CO-covered Ru(0001)^42^ or Cu(111) in a benzotriazole solution.^43^ The adsorbate-induced or surfactant-induced smoothening was attributed to an enhanced mobility of the metal atoms. While chemical Ni(OH)_2_ deposition on Cu(111) leads to an overall roughening and a stabilization of Cu ad-islands, our EC-STM results at −0.3 V_RHE_ suggest that the difference in Ni(OH)_2_ binding to Cu at lower potentials, due to, e.g., an increase in negative free charge, enhances surface diffusion and mobility of the assumed Ni(OH)_2_–Cu_ad_-complexes, which induces surface annealing. However, it cannot be excluded that the observed potential-dependent morphology changes may in addition be determined by the HER itself since Cu(100)^25^ and Cu(111)^26^ have been found to reconstruct at or close to the onset of the HER in acidic media. Generally, the Ni(OH)_2_ modification leads to drastic morphological changes where the variation of the binding of Ni(OH)_2_, in combination with the change of the free charge on the electrode, seems to result in a potential-dependent restructuring. However, only coverages of 0.2 ML of Ni(OH)_2_ create a second pme close to the onset of the HER in alkaline media, where not only a mobile and dynamic surface but also a highly disordered water network is present, which facilitates the HER by lowering the energy barrier for charge movement across the interfacial water layer.

## 3 Conclusions

In summary, we have demonstrated that modifying Cu(111) with low amounts of Ni(OH)_2_ leads to an unusual non-linear trend in the enhancement of the HER in alkaline media with increasing coverage, which is in remarkable contrast to the Ni(OH)_2_-modified Pt electrode characteristics, where a linear dependence of activity and Ni(OH)_2_ concentration is observed. Adding only 0.1 ML of Ni(OH)_2_ to the Cu surface does not lead to a significant activity increase in the HER, whereas for Ni(OH)_2_ coverages of 0.2 ML, the reaction is enhanced by a factor of 13 relative to the bare Cu(111). While there is no difference in the apparent oxidation state of Cu on the surface, as determined by XPS, *in situ* EC-STM reveals that upon Ni(OH)_2_ deposition, the surface drastically restructures, which leads to Cu ad-island nucleation and growth in the case of 0.1 ML of Ni(OH)_2_ and even a 3D roughening of the surface for 0.2 ML. This generic instability of Cu surfaces gives rise to a completely different molecular-level picture, which does not corroborate the previously found cluster-based mechanism on Pt electrodes, where Ni(OH)_2_ forms distinct nanometer-sized 3D structures serving as active sites for an enhanced water dissociation. Surprisingly, the roughness of the Cu surfaces continuously increases upon adding more Ni(OH)_2_ and thus does not clearly correlate with the observed non-linear activity trend. Laser-induced potential transients, however, reveal that only for Cu(111) modified with 0.2 ML of Ni(OH)_2_ a second pme exists, which points to the presence of a highly disordered water ad-layer close to the onset of the HER, whereas there is no significant decrease in the electric field strength for 0.1 ML of Ni(OH)_2_ compared to Cu(111). Our results therefore highlight the significant role of the interfacial water network for the electrocatalytic performance of a material, where an increase in disorder of the water ad-layer promotes charge transfer through the double layer, which drastically enhances the efficiency of the HER.

## 4 Experimental Methods

### 4.1 Chemicals and Materials

NaOH electrolyte solutions (0.1 M) were prepared by using sodium hydroxide monohydrate (Merck, Suprapur) and aqueous Ni^2+^-containing solutions (0.0001 and 0.01 M nickel sulfate hexahydrate, Alfa Aesar, >99.97%, metal basis). All solutions were prepared with ultrapure water (Milli-Q purification system, >18 MΩ cm, Merck). For deaerating the electrolytes, Ar gas (Messer, 5.0) was used. The Cu(111) single-crystal electrodes (Mateck, Jülich) were mechanically polished with diamond paste (3, 1, and 0.25 μm, ESCIL) and subsequently electropolished in 60% H_3_PO_4_ (85% EMSURE, Merck) at 1.8 V vs a Cu counter electrode and thoroughly rinsed with Milli-Q water. Prior to the STM experiments, the crystals were additionally annealed in a homemade horizontal tube furnace under H_2_ flow after electropolishing and transferred to a glovebox (MBraun MB 200 MOD) without any contact to air.

Ni(OH)_2_ deposition was performed by immersing the freshly electropolished Cu(111) single-crystal electrodes in aqueous NiSO_4_ solution for 1 min at open circuit potential, i.e., without any external potential or current applied. By employing differently diluted Ni^2+^ solutions with concentrations of either 0.0001 or 0.01 M, two different coverages of 0.1 and 0.2 ML Ni(OH)_2_ were formed. The modified Cu(111) electrodes were rinsed with NaOH solution after deposition and immersed at 0 V vs the reversible hydrogen electrode (RHE).

### 4.2 Electrochemistry

All electrochemical experiments were performed in a three electrode configuration, where the electrochemical cell included a Teflon beaker for the alkaline electrolyte to avoid any glass contamination.^44^ A carbon rod counter and a polytetrafluoroethylene (PTFE, 60% dispersion in water, Sigma Aldrich) bound activated carbon (YP-50F, Kuraray) quasi-reference (AC-QRE), which is described in detail in ref (45), were used to perform cyclic voltammetry. All potentials were converted to the reversible hydrogen electrode (RHE) scale. The Cu(111) working electrode was examined in hanging meniscus configuration. The electrochemical measurements were performed on either an eDAQ EA161 potentiostat connected to a signal generator (PAR 173) and an eDAQ ED401 digital recorder or on a Biologic VSP 300 potentiostat.

For the online differential electrochemical mass spectrometry (DEMS) measurements, a Hiden HPR-40 mass spectrometer in combination with a commercial flow cell (PEEK, Type A, Hiden Analytical, U.K.) was used. The interface between the high vacuum and the cell was a porous PTFE membrane (Gore-Tex, thickness of 75 mm, pore diameter of 0.02 μm, and a porosity of 50%). For both the counter and reference electrodes, PTFE bound activated carbon was used.

### 4.3 Quasi *In Situ* X-ray Photoelectron Spectroscopy

X-ray photoelectron spectroscopy (XPS) characterization was performed on a MultiLab 2000 instrument (Thermo Fisher Scientific) using a hemispherical sector analyzer (Alpha 110, Thermo Fischer Scientific) and a monochromatic Al Kα X-ray source (1486.6 eV). The measurements were carried out with a take-off angle of 60° between the sample and analyzer to enhance surface sensitivity. The XPS spectra were recorded at a pass energy of 25 eV and an energy step size of 0.01 eV. Pretreatment and Ni(OH)_2_ deposition were performed inside an Ar-filled glovebox, in exactly the same way as for all the electrochemical measurements. After several voltammetric cycles, all the electrodes (Cu(111), Cu(111)/0.1 ML of Ni(OH)_2_, and Cu(111)/0.2 ML of Ni(OH)_2_) were removed from the electrolyte under potential control (0 V_RHE_) and transported to an XPS analyzing chamber in a home-built transfer cell without exposure to ambient air by the additional use of a portable glovebox attached to the transfer chamber of the XPS.^46^

### 4.4 *In Situ* Electrochemical Scanning Tunneling Microscopy

Electrochemical scanning tunneling microscopy (EC-STM) experiments were performed on a Keysight 5500 scanning probe microscope, which was placed inside an Ar-filled glovebox (MBraun MB 200 MOD) to avoid any oxygen infiltration during the measurements. A home-built polychlorotrifluoroethylene (PCTFE) EC-STM cell was used, where both counter and reference electrodes consisted of PTFE bound activated carbon.^45^ STM tips were prepared by electrochemical etching of a tungsten wire (Advent, 99.9%) and subsequent coating with Apiezon wax to avoid leak currents. The Cu(111) electrode was immersed at open circuit potential, and the formed thin native copper oxide was reduced prior to every experiment. Before imaging, cyclic voltammograms were recorded inside the EC-STM cell to ensure clean conditions and the stability of the system. The metallic surface was consistently imaged prior to any Ni(OH)_2_ deposition to ensure high surface quality. For data analysis and representation of the STM images, Gwyddion^47^ was employed.

### 4.5 Laser-Induced Temperature Jump Method

The detailed procedure of the laser-induced temperature jump technique employed in this work was described previously elsewhere.^35,37,48^ Shortly, a four-electrode cell configuration was used, where the electrolyte was filled inside a Teflon piece, which was pressed onto the quartz window and allowed for an electrolyte volume of around 3 to 4 mL.^35^ A Pd–H_2_ reference electrode and two Au auxiliary electrodes were used. A Nd-YAG laser (Brilliant B, Quantel, 532 nm) was used with a pulse duration of 5 ns and a beam diameter of 6 mm. The energy density was reduced to 20 mJ/cm^2^. After several voltammetric cycles, around 100 coulostatic potential transients were recorded and averaged with a Tektronix model TDS 3054B oscilloscope.

## Supporting Information Available

Calculation of the apparent Ni(OH)_2_ coverages, statistical evaluation of the surface roughness, 3D laser transient plots, determination of the thermal coefficients, corrections for thermodiffusion potential, and dependence of the pme with coverages (PDF)
