# Supplementary material for: Interfacial Water Structure as a Descriptor for Its Electro-Reduction on Ni(OH)2-Modified Cu(111)
Source: ACS Catal. 2021 Aug 4;11(16):10324–32. doi: 10.1021/acscatal.1c02673 (PMC8383263; doi:10.1021/acscatal.1c02673)
Supplement: Supplementary file 1 — cs1c02673_si_001.pdf [file cs1c02673_si_001.pdf]

Supporting Information:

Interfacial Water Structure as Descriptor for Its Electro-Reduction on $Ni(OH)_2$ Modified Cu(111)

Andrea Auer,[†] Francisco J. Sarabia,[‡] Daniel Winkler,[†] Christoph Griesser[†], Víctor Climent,[‡*] Juan M. Feliu[‡] and Julia Kunze-Liebhäuser[†*]

[†] *Institute of Physical Chemistry, University Innsbruck, Innrain 52c, Innsbruck, 6020, Austria*
[‡] *Instituto Universitario de Electroquímica, Universidad de Alicante, Carretera San Vicente del Raspeig s/n, E-03690, San Vicente del Raspeig, Alicante, Spain*

* Corresponding author e-mail: julia.kunze@uibk.ac.at (J. Kunze-Liebhäuser)

* Corresponding author e-mail: victor.climent@ua.es (V. Climent)

Calculation of the apparent $Ni(OH)_2$ coverages

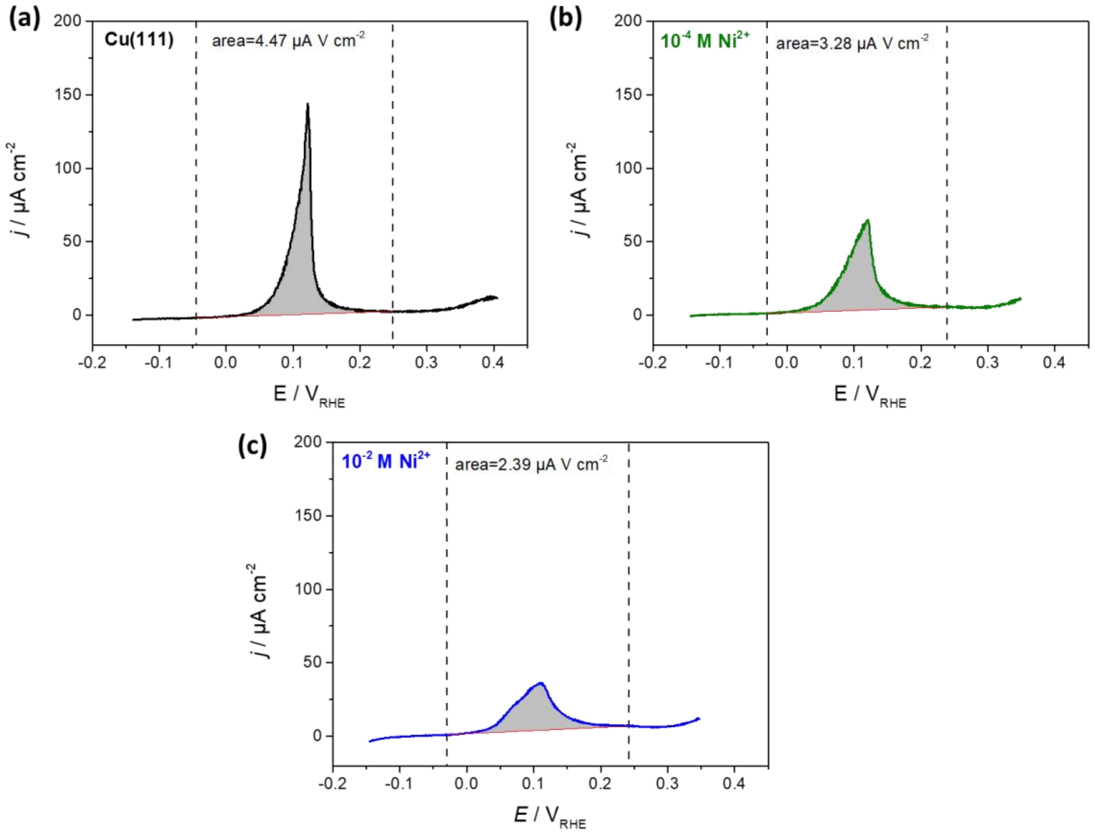

Figure S1. Determination of the apparent $Ni(OH)_2$ coverages from integration of the remaining OH adsorption peak at 0.12 V_{RHE}. Scan rate: 50 mV/s.

Since the distinct voltammetric feature, corresponding to the OH adsorption on Cu(111) at around 0.12 V_{RHE}, decreases with increasing concentration of the Ni^{2+} solutions , we are able to estimate an apparent coverage from integration of the current. A full OH adsorbate layer (1 ML, i.e. 1 OH per Cu atom) corresponds to ~205 µC cm^{-2}, i.e. 1.28 x 10^5 atoms/cm^2, if considering the previously found reconstructed Cu surface.[1] These considerations yield approximately 0.12 ML $Ni(OH)_2$ when using a 10^{-4} M concentrated $NiSO_4$ solution and 0.2 ML $Ni(OH)_2$ when using 10^{-2} M $NiSO_4$. Detailed calculations are given in Table S1.

Table S1. Integration of the OH adsorption peak.

sample	area / µA V cm^{-2}	OH adsorption charge / µC cm^{-2}	OH coverage / ML	$Ni(OH)_2$ coverage / ML
**Cu(111)**	4.5	90	0.44	–
**10^{-4} M $NiSO_4$**	3.3	66	0.32	0.12
**10^{-2} M $NiSO_4$**	2.4	48	0.23	0.20

Statistical evaluation of the surface roughness

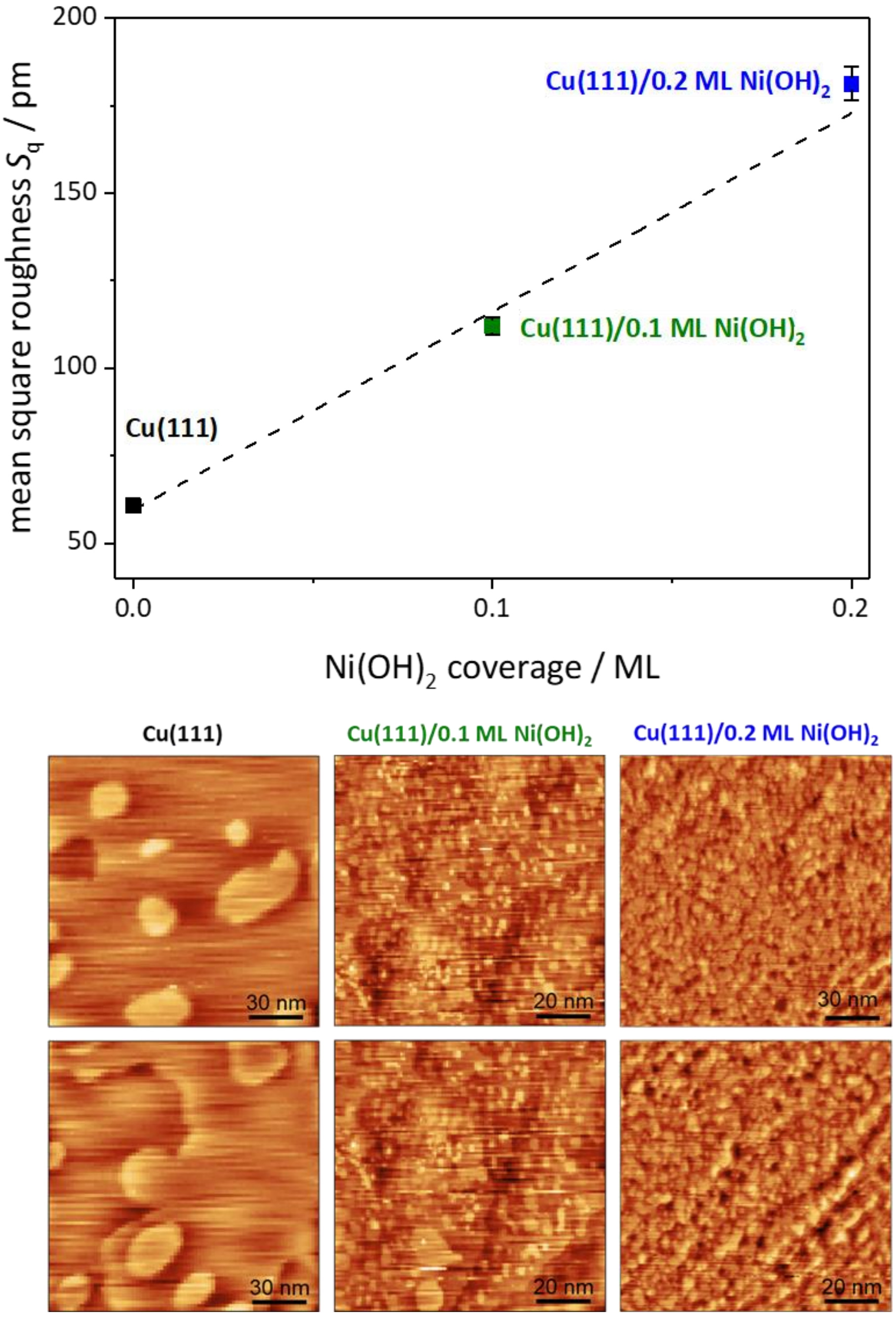

Figure S2. Statistical evaluation of the surface roughness.

The mean square roughness S_q was determined for three different positions on the surfaces (two are exemplarily given in Figure S2), using the corresponding tool in Gwyddion.[2] Plotting the average values for S_q versus the $Ni(OH)_2$ coverage results in a linear dependence. This suggests that, while the increase in activity towards the HER scales non-linearly with higher coverages (from 0.1 to 0.2 ML $Ni(OH)_2$), the surface roughness in fact increases continuously.

Table S2. Values of the mean square surface roughness. Average values and errors are determined by evaluation of three different positions on the surfaces.

sample	Average S_q / pm	Error of the average value / pm
**Cu(111)**	60.7	1.9
**10^{-4} M $NiSO_4$**	112.0	2.4
**10^{-2} M $NiSO_4$**	181.3	4.8

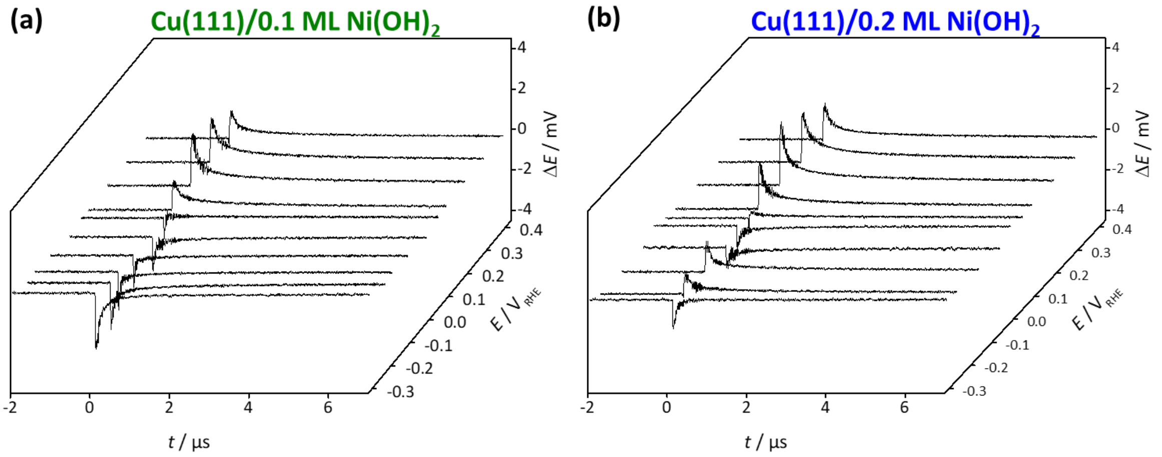

Figure S3. 3D plots of the laser transients (ΔE vs t) recorded on Cu(111) over the potential range between -0.3 and 0.4 V_{RHE} with different $Ni(OH)_2$ coverages. (a) 0.1 ML $Ni(OH)_2$ and (b) 0.2 ML $Ni(OH)_2$.

Determination of the thermal coefficients from the laser transients

Generally, in the absence of specific adsorption phenomena or when their responses are negligible, the potential change ΔE recorded after the laser pulse follows the relaxation of the temperature at the interface. Assuming that the non-reflected part of the irradiated laser beam is suddenly converted into heat, the change of temperature with time can be described as follows[3,4]:

$$\Delta T = \frac{2(1-R)I}{\sqrt{\pi}}\left(\frac{\kappa}{\sqrt{\alpha}}+\frac{\kappa_1}{\sqrt{\alpha_1}}\right)^{-1}\left(\sqrt{t}-\sqrt{t-t_0}\right),\ t > t_0 \tag{1}$$

Where the maximum temperature change at the surface (at t=t_0=5ns) is:

$$\Delta T_0 = \frac{2(1-R)I}{\sqrt{\pi}}\left(\frac{\kappa}{\sqrt{\alpha}}+\frac{\kappa_1}{\sqrt{\alpha_1}}\right)^{-1}\sqrt{t_0} \tag{2}$$

R is the reflectivity of the surface, I the laser intensity, κ,α and κ_1,α_1 are the thermal conductivity and the thermal diffusivity of the metal and the solution, respectively, where $\alpha = \kappa/\rho C$ with ρ the material density and C the heat capacity.

The heat flux on the metal (q) can be described as:

$$q = (1-R)I \tag{3}$$

Using the thermal and optical constant for copper (Table S3), we can estimate the maximum change of temperature to be 28 K. Note that we cannot account for possible variations of these constants upon $Ni(OH)_2$ deposition. A beam energy I of 3.54 $MWcm^{-2}$ was used.

Table S3. Optical and thermal constants for Cu and water. From Refs. [5,6].

	ρ / (g cm^{-3})	κ / (J cm^{-1} K^{-1} s^{-1})	C / J g^{-1} K^{-1}	R (at 532 nm)
**Cu**	8.96	3.85	0.386	0.627
**Water**	1	0.006	4.19	–

With the maximum temperature change (ΔT_0 = 28 K), we can now determine the thermal coefficient.

The temperature decay for sufficiently long time (t>>t_0) follows:

$$\Delta T = \frac{1}{2}\Delta T_0\sqrt{\frac{t_0}{t}} \tag{4}$$

Since the change of the electrode potential (ΔE) follows the change of temperature, it can be described as:

$$\Delta E = \left(\frac{\partial E}{\partial T}\right)_q \Delta T = \frac{1}{2}\left(\frac{\partial E}{\partial T}\right)_q \Delta T_0 \sqrt{\frac{t_0}{t}} \tag{5}$$

Where $\left(\frac{\partial E}{\partial T}\right)_q$ is the thermal coefficient at any given potential and q is the charge density on the metal. We can easily extract these coefficients from the slopes obtained after linearization of the transients (ΔE vs $1/\sqrt{t}$), shown in Figure S5, using the following equation:

$$\left(\frac{\partial E}{\partial T}\right)_q = \frac{2 \cdot slope}{\Delta T_0 \sqrt{t_0}} \tag{6}$$

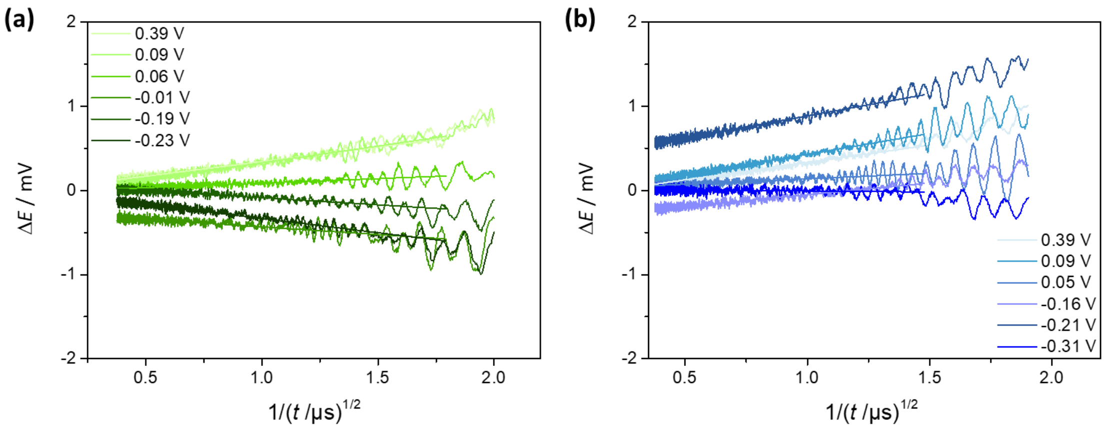

Figure S4. Linearization of the laser-induced potential transients for determination of the thermal coefficients. (a) 0.1 ML $Ni(OH)_2$ and (b) 0.2 ML $Ni(OH)_2$.

The potential of zero response (pzr), where $\left(\frac{\partial E}{\partial T}\right)_q = 0$, corresponds to a maximum disorder in the interfacial water layer according to the thermodynamic relation between the thermal coefficient and the entropy of formation of the double layer[7,8]:

$$\left(\frac{\partial E}{\partial T}\right)_q = -\left(\frac{\partial(\Delta S_{dl})}{\partial q}\right)_T \tag{7}$$

ΔS_{dl} is defined as the difference in the entropy of the double layer components when they are present in the formed interface and in the bulk of the adjoining phases.[7]

The pzr, i.e. the pme, is hence the potential at which water molecules distribute randomly and there is no net dipolar contribution to the electrode potential. Therefore, it is closely related to the potential of zero free charge, since water dipoles orient according to the electric field at the surface.

Correction for the thermodiffusion potential

The thermodiffusion potential arises from the temperature differences between the solution surrounding the reference and the working electrode, which causes a potential drop due to the motion of ions resulting from the thermal gradient. While in most cases such contributions can be neglected, in the case of highly alkaline solutions, due to the abnormally high mobility of OH^- ions, uncorrected thermal coefficients are slightly overestimated (see Figure S6).

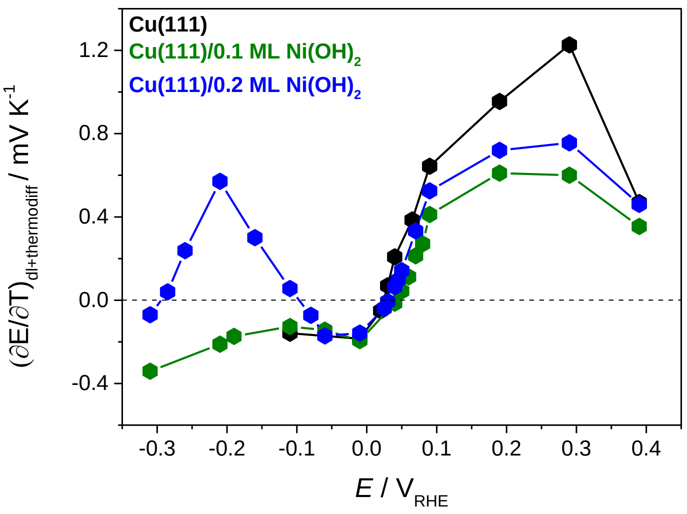

Figure S5. Thermal coefficients (∂E/∂T) without correction for the thermodiffusion potential.

To provide more accurate results, we therefore need to estimate the thermodiffusion potential from the Eastman entropies of transport and mobility of ions[9]. This results in an approximate value of −0.43 mV K^{-1} in 0.1 M NaOH,[10] which is then used to obtain the corrected thermal coefficients shown in Figure 5 in the main text.

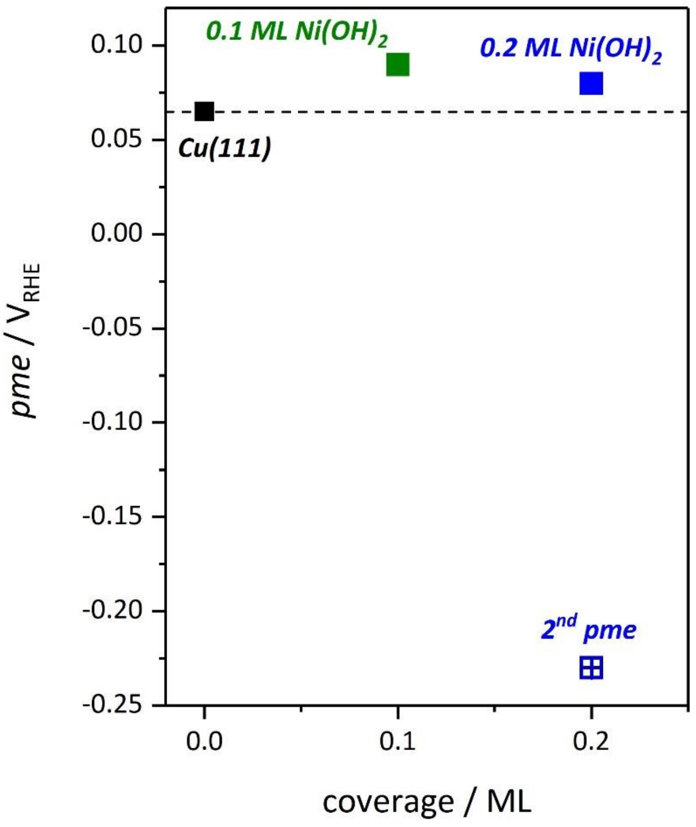

Figure S6 Dependence of the potential of maximum entropy on the $Ni(OH)_2$ coverage. Color legend: Cu(111) (black) modified with 0.1 ML $Ni(OH)_2$ (green) and 0.2 ML $Ni(OH)_2$ (blue).

In Figure S6 the extracted values for the potentials of maximum entropy of Cu(111), Cu(111)/0.1 ML $Ni(OH)_2$ and Cu(111)/0.2 ML $Ni(OH)_2$ are shown. The pme was determined by extracting the position where $\left(\frac{\partial E}{\partial T}\right) = 0$. It has to be noted that in the case of 0.2 ML of $Ni(OH)_2$ there are three potentials at which the thermal coefficient becomes zero, however, only the change of sign going from negative to positive can be considered as a second pme, according to the integration of equation (7).

Supporting References

(1) Auer, A.; Ding, X.; Bandarenka, A. S.; Kunze-Liebhäuser, J. The Potential of Zero Charge and the Electrochemical Interface Structure of Cu(111) in Alkaline Solutions. *J. Phys. Chem. C* **2021**, *125* (9), 5020–5028. DOI: 10.1021/acs.jpcc.0c09289

(2) Necas, D.; Klapetek, P. Gwyddion: An Open-Source Software for SPM Data Analysis. *Centr. Eur. J. Phys.* **2012**, *10*, 181–188. DOI: 0.2478/s11534-011-0096-2

(3) Benderskii, V. A.; Velichko, G. I. Temperature Jump in Electric Double-Layer Study. Part I. Method of Measurements. *J. Electroanal. Chem.* **1982**, *140* (1), 1–22. DOI: 10.1016/0368-1874(82)85295-7

(4) Climent, V.; Coles, B. A.; Compton, R. G. Laser-Induced Potential Transients on a Au(111) Single-Crystal Electrode. Determination of the Potential of Maximum Entropy of Double-Layer Formation. *J. Phys. Chem. B* **2002**, *106* (20), 5258–5265. DOI: 10.1021/jp020054q

(5) Weber, M. J. *Handbook of Optical Materials*; CRC Press LLC, 2003.

(6) Nave, R. *Hyperphysics*. http://hyperphysics.phy-astr.gsu.edu (accessed 2021-04-13).

(7) Harrison, J. A.; Randles, J. E. B.; Schiffrin, D. J. The Entropy of Formation of the Mercury-Aqueous Solution Interface and the Structure of the Inner Layer. *J. Electroanal. Chem.* **1973**, *48* (3), 359–381. DOI: 10.1016/S0022-0728(73)80369-9

(8) García-Aráez, N.; Climent, V.; Feliu, J. M. Evidence of Water Reorientation on Model Electrocatalytic Surfaces from Nanosecond-Laser-Pulsed Experiments. *J. Am. Chem. Soc.* **2008**, *130* (12), 3824–3833. DOI: 10.1021/ja0761481

(9) Agar, J. N. *Thermogalvanic Cells*, Vol. 3.; Wiley-Interscience: New York, 1963.

(10) García-Aráez, N.; Climent, V.; Feliu, J. M. Temperature Effects on Platinum Single-Crystal/Aqueous Solution Interhphases. Combining Gibbs Thermodynamics with Laser-Pulsed Experiments. In *Interfacial Phenomena in Electrocatalysis*; Vayenas, C. G., Ed.; Springer Science+Business Media, 2011; pp 1–106.
